# 

**Published:** 1854-03

**Authors:** 


### RECEIPTS

### FOR THE JOURNAL DURING THE MONTH OF MARCH.

**Illinois.**

| | | |
|---|---|---|
| Dr | L. C. White, | $2 00 |
| " | N. E. Niglers, | 2 00 |
| " | A. Mahan, | 2 00 |
| " | C. Wheeler, | 2 00 |
| " | Hammill, | 2 00 |
| " | B A Fuller, | 2 00 |
| " | W. B. Simonton. | 2 00 |
| " | R. C. Edgerton, | 2 00 |
| " | E. C. Ellet, | 2 00 |
| " | L. A. Mease, | 2 00 |
| " | Dr. Gorham, | 4 00 |
| " | John Allen, | 2 00 |
| " | Durfee & Baldwin, | 4 00 |
| " | I. G. Tilden, | 2 00 |
| " | R. C. Muir, | 2 00 |

**Michigan.**

| | | |
|---|---|---|
| Dr. | S. P. Root, | 2 00 |
| " | J. S. Skinner, | 1 00 |

**Indiana.**

| | | |
|---|---|---|
| Dr. | F. Stevens, | $2 00 |
| " | J. G. Johnson, | 2 00 |
| " | R. J. Simpkins. | 2 00 |
| " | R. M. Gilkeson, | 1 00 |
| " | G. W. Slack, | 2 00 |
| " | Samuel Riefenbrick, | 2 00 |
| " | T. A. Graham, | 1 00 |

**Wisconsin.**

| | | |
|---|---|---|
| Dr. | W. H. Smith, | $2 00 |
| " | L. R. Brown, | 2 00 |
| " | John D, Wood, | 2 00 |
| " | J. M. Evans, | 6 00 |
| " | Hopkins, | 2 00 |
| " | H. Warne, | 2 00 |
| " | Clark & Rice, | 2 00 |

**Iowa**

| | | |
|---|---|---|
| Dr. | R. C. Ford, | $4 00 |

